# Met-enkephalin modulates the stress responses of plasma concentrations of corticosterone, delta opioid receptor binding, pro-enkephalin expression, and processing in chickens

**DOI:** 10.3389/fphys.2025.1736176

**Published:** 2026-01-26

**Authors:** Krystyna Pierzchała-Koziec, Colin G. Scanes, Klaudia Jaszcza

**Affiliations:** 1 Department of Animal Physiology and Endocrinology, University of Agriculture in Krakow, Krakow, Poland; 2 Department of Biological Science, University of Wisconsin Milwaukee, Milwaukee, WI, United States

**Keywords:** chicken, corticosterone, Met-enkephalin, pro-enkephalin, stress response

## Abstract

**Introduction:**

Met-enkephalin is a neuropeptide whose release into the circulation is enhanced by stress. There have been no studies on the effects of peripherally administered Met-enkephalin in chickens.

**Methods:**

The effects of peripheral administration of Met-enkephalin on the stress response in chickens were investigated measuring plasma concentrations of corticosterone and Met-enkephalin, together with expression of pro-enkephalin (PENK) and delta-opioid binding in the hypothalamus, anterior pituitary and adrenal glands.

**Results:**

Administration of Met-enkephalin was followed by decreases in the basal and stressed plasma concentrations of the principal glucocorticoid, corticosterone, in chickens. In addition, the increase in plasma concentrations of corticosterone evoked by restraint stress was markedly decreased when the birds were treated with Met-enkephalin. Administration of Met-enkephalin was followed by decreases in PENK expression; hypothalamic, anterior pituitary, and adrenal delta-opioid binding; and plasma concentrations of total Met-enkephalin (peptides containing Met-enkephalin motifs). There were negative relationships between plasma concentrations of corticosterone and Met-enkephalin and between those of native Met-enkephalin and total Met-enkephalin.

**Discussion:**

The ability of Met-enkephalin to attenuate the stress response of corticosterone, and probably other glucocorticoids, is novel and opens up several new lines of inquiry, including its site of action and its source.

## 1 Introduction

Met-enkephalin exists in both the blood and tissues in two forms: native Met-enkephalin with five amino acid residues (YGGFL) and total Met-enkephalin; the latter represents either precursor peptides containing Met-enkephalin motifs or Met-enkephalin associated with large proteins (Pierzchala and Van Loon, 1990). Endogenous opioid peptides modulate the hypothalamic–pituitary–adrenal axis (HPA) activity (Drolet et al., 2001; Russell et al., 2008). Opioid-containing neurons innervate the median eminence and paraventricular nucleus of the hypothalamus, regulating the release of corticotropin-releasing hormone (Stein and Zöllner, 2009; Di et al., 2007).

Plasma concentrations of both corticosterone and Met-enkephalin are increased in young female chickens after subjecting young chickens to a series of stresses such as restraint stress, crowding, withholding water, or withholding feed (Scanes and Pierzchala-Koziec, 2024a; Scanes et al., 2024). This is consistent with corticosterone, together with Met-enkephalin, being constituents of the hypothalamic–pituitary–adrenal axis. In contrast, although peripheral administration of morphine is followed by increases in plasma concentrations of corticosterone, there are decreased plasma and HPA tissue concentrations of Met-enkephalin together with adrenal expression of pro-enkephalin (PENK) in young female chickens (Scanes and Pierzchala-Koziec, 2024b). The morphine-induced elevation in plasma concentrations of corticosterone, indicating activation of the HPA axis, is similar to that observed in other species and is consistent with morphine acting centrally to activate the HPA cascade (Scanes and Pierzchala-Koziec, 2024b). The decrease in plasma and tissue concentrations of Met-enkephalin together with PENK expression following morphine administration is explicable with central or peripheral effects or negative feedback locally or systemically. The opioid agonist, morphine, acts both peripherally and centrally (Cunha et al., 2010) and via binding to the delta (δ) opioid receptor (DOR), the kappa (κ) receptor (KOR), and the mu (μ) opioid receptor (MOR) (Dhaliwal and Gupta, 2025).

It is hypothesized that peripherally administered Met-enkephalin would have effects at the levels of the corticotropes or the adrenal tissues but probably not the hypothalamus, due to the blood–brain barrier. An exhaustive review of the literature indicated that there are no reports on the effects of peripherally administered Met-enkephalin in chickens or on the effects of Met-enkephalin on the HPA axis in any species. The present study examines the effects of peripherally administered Met-enkephalin on plasma and adrenal corticosterone concentrations, plasma Met-enkephalin concentrations, and adrenal PENK expression in chickens. It is noted that the half-life of Met-enkephalin following i.v. administration is widely assumed to be very short; for instance, the half-life of Met-enkephalin was reported to be less than 2 min (Hartvig et al., 1986). A half-life of 3.9 min was reported after ventricular administration (Craves et al., 1978). In contrast, PubChem reports a half-life for Met-enkephalin ranging from 4.2 to 39 min. The half-life for Met-enkephalin is 3–4 days in cultured chromaffin cells (Wilson, 1991). Moreover, it was hypothesized that administration of Met-enkephalin would downregulate DOR in the pituitary and adrenal glands. This was also examined in the present study.

## 2 Materials and methods

All animal procedures were conducted with prior institutional ethical approval in accordance with the Local Institutional Animal Care and Use Committee (IACUC) and followed ARRIVE guidelines. The chicken study protocol 120/2013 was approved by the Institutional Review Board and the First Local Ethical Committee on Animal Testing in Krakow, Poland.

### 2.1 Animals

The study used 20 female, 14-week-old ISA Brown hybrid (Rhode Island x Leghorn) chickens weighing 1.2 ± 0.10 kg. The age was chosen to be prior to sexual maturation in the birds (Hrabia et al., 2011). The birds were maintained in individual cages (60 × 60 × 60 cm) in a controlled environment (photoperiod 12L/12D, with lights on from 7 a.m. to 7 p.m.) at room temperature (20 °C). The chickens received feed and water *ad libitum*. The animals were habituated to these conditions for 7 days before experimentation.

### 2.2 Experimental design

**FIGURE 1:**
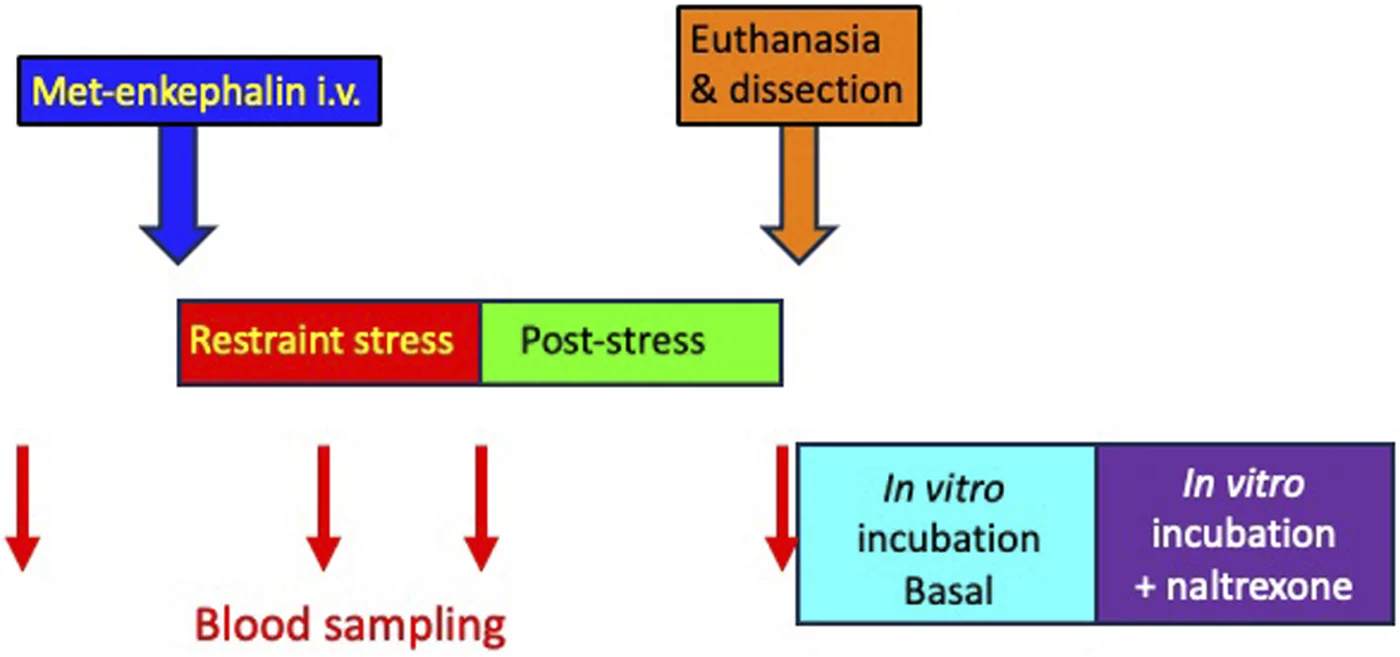
Schematic representation of the experimental design.

Figure 1 provides a schematic representation of the experimental design. Twenty 14-week-old female chickens were randomly assigned to four treatment groups (n = 5 per group): 1) control (saline vehicle injected i.v.); 2) i.v. injection of Met-enkephalin; 3) stressed (plus saline vehicle injected i.v.), and 4) injected with Met-enkephalin and stressed.

Pretreatment blood samples were collected, after which the chickens received an i.v. injection of either 0.9% saline or Met-enkephalin (1 mg/kg b.wt.). The chickens were subjected to restraint stress by placing them in boxes (30 × 30 × 30 cm) without light and sound for 30 min. To collect the blood samples, the birds were transitorily manually held, and the samples (each of 2 mL) were taken from the left wing-vein and transferred into heparinized tubes (corticosterone) containing EDTA (5%), citric acid (17.7 mol mL^-1^), and aprotinin (Trasylol, 200 KIU/mL)] for Met-enkephalin determination under conditions where degradation of Met-enkephalin was inhibited. Blood samples were taken at the following times: 15 min and 1 min before the initiation of the stress or Met-enkephalin or vehicle, 15 and 30 min after the initiation of restraint, and 30 min after terminating the stress (Figure 1). The sampling times were based on previous studies in which restraint stress in chickens was accompanied within 15 min by increased plasma concentrations of corticosterone (Kadhim and Kuenzel, 2022; Scanes et al., 2024). The blood samples were taken at the same times, starting at 9 a.m. Blood samples were centrifuged for 30 min at 4 °C and 4,000 x g, and the plasma was immediately frozen at −80 °C until further processing.

Immediately after the last blood sample was collected, the chickens were euthanized by i.v. injection of 70 mg/kg m.c. of pentobarbital (Exagon, Richter Pharma AG) according to the NIH (2020) guidelines. Tissues (hypothalamus, pituitary, and adrenals) were dissected and used for gene expression analysis (freezing to −80 °C), receptor binding (homogenized), and hormone secretion assays (placed in cold buffer).

### 2.3 Hormone assays

The concentrations of Met-enkephalin (native and total) in the plasma (100 µL) were determined by radioimmunoassay using the method proposed by Pierzchała-Koziec et al. (2019). In brief, enkephalin-containing peptides (total enkephalin) were hydrolyzed with trypsin and carboxypeptidase B. Optimal conditions for hydrolysis of the total enkephalins included incubation with trypsin (1 mg/mL, 37 °C) for 30 min, followed by incubation with carboxypeptidase B (5 mg/mL) plus trypsin inhibitors (2.5 mg/mL) for 15 min.

Native and total enkephalins were purified on Porapak Q (Waters, 100,120 mesh) in 2 mL of absolute ethanol, then lyophilized, and subjected to radioimmunoassay. Met-enkephalin immunoreactivity was quantified using a commercial antiserum developed in rabbit, ^125^I-Met-enkephalin, and a Met-enkephalin standard. Lyophilized samples were reconstituted with 100 µL of 0.06 M phosphate buffer (pH 6.5; 0.2% bovine serum albumin and 0.002% sodium azide). Then, 50 µL of antiserum (1:10,000) and 50 µL of ^125^I-Met-enkephalin (∼1,500 cpm) were added, and the samples were incubated (4 °C). After 24 h, 50 µL of rabbit γ-globulin (1%) was added, and incubation was maintained for 30 min. Bound and free complexes were separated by adding 250 µL of 25% polyethylene glycol (PEG 8000). After 30 min of incubation, samples were centrifuged (2000 ×g, 4 °C, 20 min); the supernatants were discarded, and the pellets were counted in a γ-counter (Wizard). The inter-assay and intra-assay coefficients of variance were, respectively, 7% and 11%.

The circulating levels of corticosterone were measured in duplicate using a radioimmunoassay (RIA) with a Corticosterone Double Antibody RIA Kit (07–120102, MP Biomedicals, Irvine, CA, United States) using 10 µL of plasma. The intra-assay and inter-assay coefficients of variance were 9.1% and 15.5%, respectively.

### 2.4 Delta opioid receptor binding

Delta opioid receptor binding was determined using the method proposed by Belcheva et al. (1994) and Hytrek et al. (1996), with some modification (Pierzchała-Koziec et al., 2017). In brief, the dissected tissues (hypothalamus, pituitary, and adrenal) were homogenized in ice-cold buffer 50 mM Tris-HCl at pH 7.4, and the homogenate was centrifuged at 20,000 x g for 15 min. Cell membrane preparations (1 mL, 1 mg of protein) were incubated at 30 °C for 30 min with the 6.80 nM titrated δ-opioid receptor agonist D-Ala^2^-N-Me-Phe^4^-Gly-ol (Amersham International). Nonspecific binding was estimated with 10 μM of unlabeled Met-enkephalin (Sigma, St. Louis, MO, United States). Free ligand was separated from the membrane-bound radioligand through filtration under reduced pressure using GF/B Whatman glass filters.

Protein concentrations in tissues were determined using the bicinchoninic acid (BCA) method (Olson and Markwell, 2007).

### 2.5 Pro-enkephalin mRNA gene expression

Pro-enkephalin gene expression was estimated by a modification of the *in situ* hybridization method proposed by Lightman and Young (1987). *In situ* hybridization is a laboratory technique used to localize a sequence of DNA or RNA in a biological sample. In this technique, a biological sample consisting of chromosomes from an individual is affixed to a glass slide and then exposed to a “probe”—a small piece of single-stranded DNA tagged with an isotope. The labeled probe finds and then binds to its matching sequence within the biological sample.

In brief, the frozen adrenals were sectioned (14 µm sections) using a Leica cryostat microtome (−22 °C). From the left adrenal gland, 15 slices were collected: from the anterior (5), middle (5), and posterior (5) parts. The sections/slices were thaw-mounted on gelatin-covered microscopic slides and stored for 3 days at −20 °C before the assay. Then, tissue sections were thawed and fixed in 4% formaldehyde in phosphate-buffered saline (PBS; pH 7.4) for 10 min. Then, sections were acylated for 10 min in triethanolamine/acetic anhydride (0.25%). Sections were dehydrated by immersion through graded ethanol (70%, 80%, 95%, and 100%) and air-dried. After pre-hybridization, a synthetic deoxyoligonucleotide, complementary to the fragment of rats or hens, PENK, was labeled using ^35^S-dATP (1,200 Ci nmol^-1^) to obtain a specific activity of approximately 4 × 10^6^ cpm/μL. The probes were diluted in a hybridization buffer [formamide, dextran sulfate, saline–sodium citrate (SSC) buffer, Denhardt’s solution, yeast tRNA, and herring sperm DNA]. Hybridization occurred for 20 h in a humidified chamber at 37 °C. The sections were then washed once in SSC for 10 min, followed by four washes of 15 min each in SSC/50% formamide at 40 °C, rinsed in SSC and distilled water at room temperature, and air-dried. The sections were exposed to Kodak film for 4 weeks (−80 °C). The photo-stimulated luminescence (PSL) density of the irradiated plates was measured using a BAS-1000 readout system. Optical density was measured at 20 locations in each slice, resulting in 300 readings per adrenal, which were subsequently subjected to statistical analysis. A computer image analysis system was used to determine PSL/mm^2^ in film images.

### 2.6 Corticosterone secretion

*In vitro* corticosterone secretion from adrenal fragments was measured according to the method proposed by Kowalski and Giraud (1993), with some modifications. In brief, fragments of tissues (20–30 mg) sliced using a microtome were placed into 24-well plates with 1 mL of Krebs–Ringer bicarbonate buffer (medium). After a 20-min pre-incubation period, tissues were incubated at 37 °C for 30 min in 1 mL of medium (basal); then, 100 nM of naltrexone (an opioid receptor antagonist) was added for the next 30 min. Media were collected and stored at −80 °C until estimation of corticosterone.

### 2.7 Statistical analysis

Time-series data from the same animals were analyzed using repeated-measures ANOVA. Data on the effects of the treatments at different time points or expressed as delta area under the curve (following treatment) minus the initial concentration were analyzed by two-way ANOVA, with *p* < 0.05 considered significant. Means were compared using Tukey’s honestly significant difference test.

## 3 Results

The effects of *in vivo* administration of Met-enkephalin or restraint stress on plasma concentrations of corticosterone, native Met-enkephalin, and total Met-enkephalin were examined.

**FIGURE 2:**
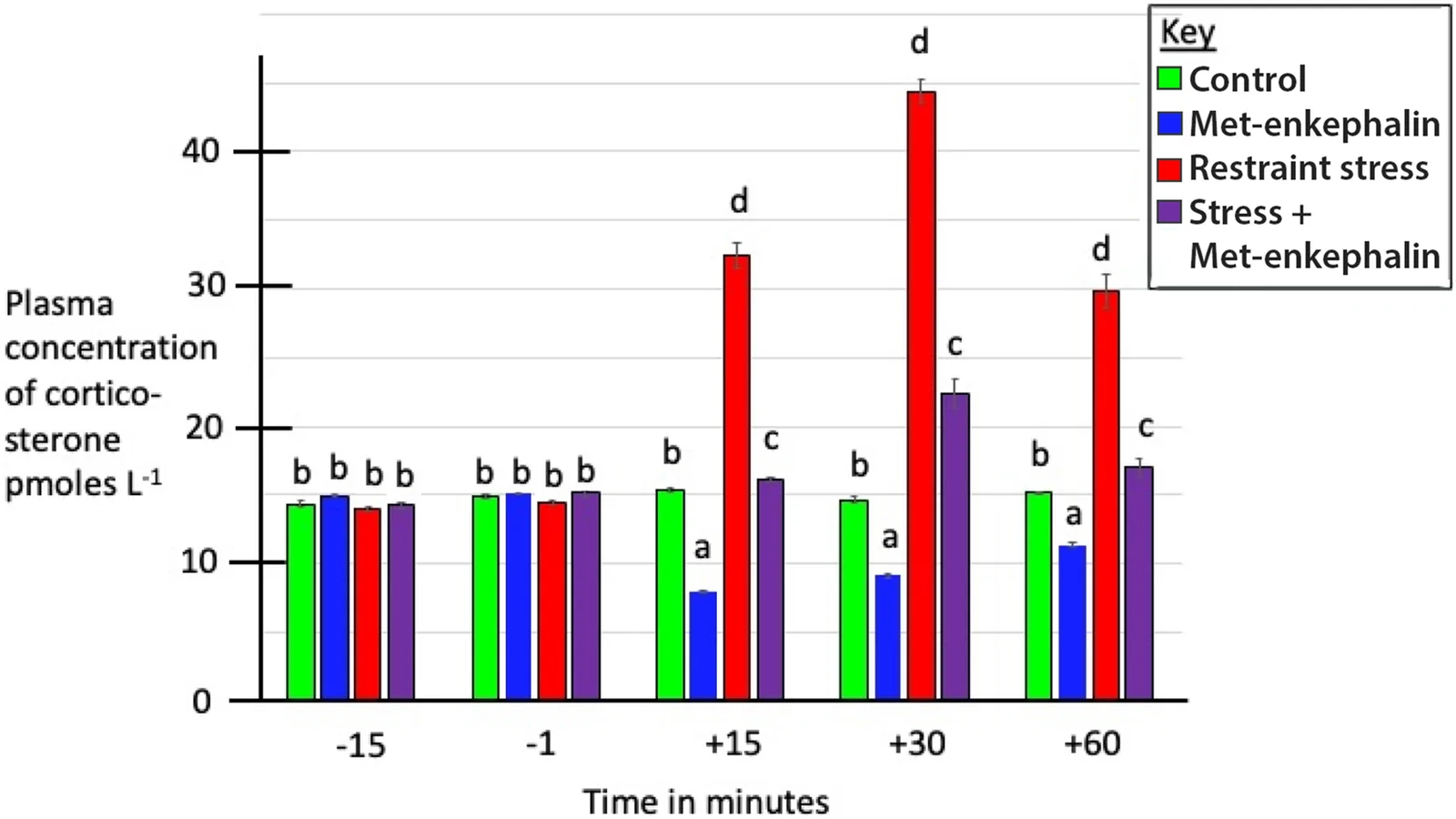
Effects of peripheral administration of Met-enkephalin or restraint stress on plasma concentrations of corticosterone (pmol per L, mean ± SEM) [vertical lines indicate SEM. Different letters indicate difference (*p* < 0.001)].

Plasma concentrations of corticosterone were increased (*p* < 0.001) in pullets subjected to restraint stress (Figure 2; Table 1). In contrast, plasma concentrations of corticosterone were depressed (*p* < 0.001) following Met-enkephalin administration. Moreover, the increase in plasma concentrations of corticosterone in stressed chickens was markedly attenuated by Met-enkephalin administration (Figure 2).

**TABLE 1:** Effect of *in vivo* administration of Met-enkephalin or restraint stress on plasma concentrations of corticosterone as area under the curve (pmol·per L·x time in min), native Met-enkephalin (pg·per ml·x time in min), total Met-enkephalin including native Met-enkephalin (pg·per ml·x time in min), and total Met-enkephalin excluding native Met-enkephalin in 14-week-old (sexually immature) female chickens (X ± SEM).

*In vivo* treatment	Plasma concentration as area under the curve minus initial			
	Corticosterone	Native met-enkephalin	Total met-enkephalin	
			Including native met-enkephalin	Excluding native met-enkephalin
Vehicle	18.9 ± 2.8^b^	−76.3 ± 19.2^a^	−3,150 ± 868^a^	−3,226 ± 881^a^
Met-enkephalin	−337 ± 5.0^a^	616 ± 41.2^b^	−3,810 ± 1120^a^	−4,426 ± 1097^a^
Stress	1,414 ± 13.9^d^	1,180 ± 29.4^c^	−12165 ± 311^c^	−13345 ± 324^b^
Met-enkephalin + stress	289 ± 47.6^c^	1,344 ± 50.1^day^	−9,900 ± 756^b^	−11,244 ± 767^b^
Two-way ANOVA F = (*p* =)				
Effect of Met-enkephalin	**879 (2.07E** ^**15**^ **)**	**135 (3.31E** ^**-9**^ **)**	**8.23 (0.011)**	**11.5 (0.0004)**
Effect of stress	**1,636 (1.53E** ^**-17**^ **)**	**724 (9.49E** ^**-15**^ **)**	**171 (5.83E** ^**-10**^ **)**	**205 (1.55E** ^**-10**^ **)**
Interaction	**237 (5.23E** ^**-11**^ **)**	**51.5 (2.20** ^**E-6**^ **)**	**31.8 (3.70E** ^**-5**^ **)**	**35.6 (1.97E** ^**-5**^ **)**

a, b, c, and d: Different superscript letters indicate differences (*p*< 0.05) with *in vivo* treatment (columns).

Bold indicates statistically significant (*P* < 0.05).

Responses to *in vivo* administration of Met-enkephalin or restraint stress were estimated by the increased/decreased area under the curve for 60 min following treatment [(concentration in ng mL^−^ − the initial concentration^−1^) × time in minutes)]. Plasma concentrations of corticosterone (area under the curve) were decreased (*p* < 0.001) in pullets receiving administration of native Met-enkephalin and increased (*p* < 0.001) in stressed birds (Table 1). As would be expected, plasma concentrations of native Met-enkephalin (area under the curve) were elevated (*p* < 0.001) in chickens receiving treatment with Met-enkephalin. There were increases (*p* < 0.001) in plasma concentrations of native Met-enkephalin (area under the curve) in chickens subjected to restraint stress (Table 1). In contrast to the increases in the plasma concentrations of native Met-enkephalin (area under the curve) in chickens receiving Met-enkephalin injection or restraint stress (Table 1), total Met-enkephalin (area under the curve) was decreased (*p* < 0.001) in pullets treated with Met-enkephalin injection or restraint stress (Table 1).

### 3.1 Effect of *in vivo* administration of Met-enkephalin or restraint stress on DOR binding

**FIGURE 3:**
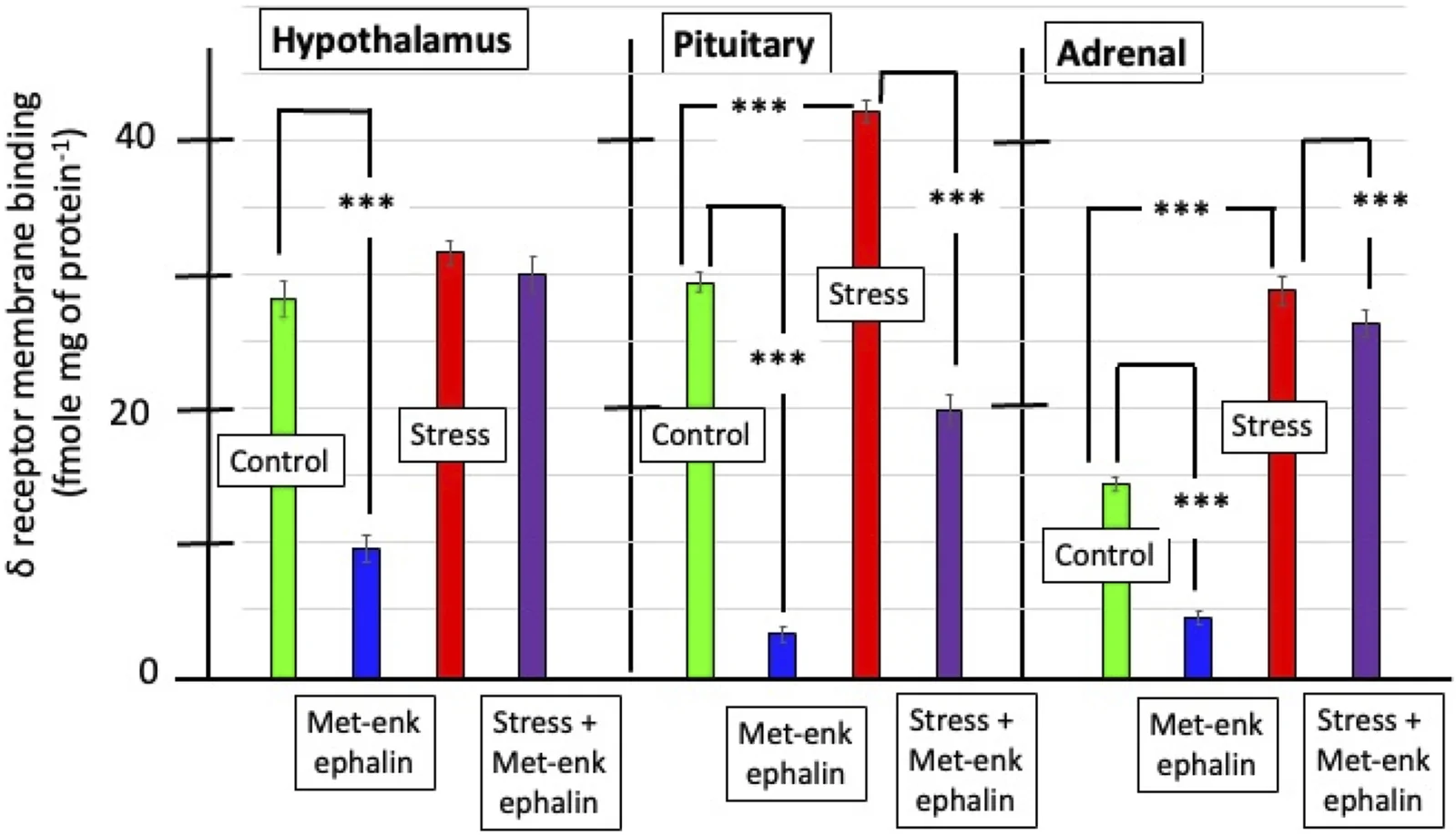
Effect of administration of Met-enkephalin or stress on delta opioid receptor binding (fmol per mg protein^−1^ in the cell membrane preparations) in the chicken hypothalamus, anterior pituitary gland, and adrenal glands (vertical lines indicate SEM). (*** indicates difference *p* < 0.001).

Administration of Met-enkephalin or restraint stress *in vivo* influenced DOR binding in tissues of the HPA axis (Figure 3). Administration of Met-enkephalin reduced (*p* < 0.001) DOR binding in hypothalamic, anterior pituitary, and adrenal tissues (Table 2) by, respectively, 66.0%, 89.1%, and 69.4%. In contrast, restraint stress was followed by increases (*p* < 0.001) in DOR binding in hypothalamic, anterior pituitary, and adrenal tissues (Table 2) by, respectively, 12.0%, 43.5%, and 100%.

**TABLE 2:** Effect of *in vivo* administration of Met-enkephalin or restraint stress on delta opioid binding in hypothalamic, anterior pituitary, and adrenal tissue in 14-week-old (sexually immature) female chickens (X ± SEM).

*In vivo* treatment	Delta opioid binding as fmol per mg of protein in the cell membrane preparations		
	Hypothalamus	Anterior pituitary gland	Adrenal
Vehicle	28.2 ± 1.39^b^	29.4 ± 0.75^c^	14.4 ± 0.51^b^
Met-enkephalin	9.6 ± 1.03^a^	3.2 ± 0.58^a^	4.4 ± 0.51^a^
Stress	31.6 ± 0.93^c^	42.2 ± 0.86^d^	28.8 ± 1.07^d^
Met-enkephalin + stress	30.0 ± 1.30^bc^	19.8 ± 1.16^b^	26.4 ± 0.93^c^
Two-way ANOVA F = (*p* =)			
Met-enkephalin	**73.4 (2.26E** ^**-7**^ **)**	**793 (4.66E** ^**-15**^ **)**	**61.0 (7.54E** ^**-7**^ **)**
Effect of stress	**102 (2.41E** ^**-8**^ **)**	**290 (1.12E** ^**-11**^ **)**	**526 (1.15E** ^**-13**^ **)**
Interaction	**52.0 (2.08E** ^**-6**^ **)**	**4.85 (0.043)**	**22.9 (0.0002)**

a, b, c, and d: Different superscript letters indicate difference (*p* < 0.05) with *in vivo* treatment (columns).

Bold indicates statistically significant (*P* < 0.05).

### 3.2 Effects of administration of Met-enkephalin or restraint stress *in vivo* on adrenal tissue

Basal *in vitro* release of corticosterone from adrenal tissue in the presence or absence of naltrexone was greater (*p* < 0.001) in adrenal tissue than in Met-enkephalin-treated pullets (Table 3). Although *in vitro* corticosterone release was decreased (*p* < 0.001) in the presence of naltrexone in adrenal cortical tissue from stressed birds, basal corticosterone release was not affected by restraint stress (Table 3). Consistent increases (*p* < 0.001) in corticosterone release in adrenal tissues incubated with the opioid antagonist naltrexone, irrespective of the *in vivo* treatments (Table 3).

**TABLE 3:** Effect of *in vivo* administration of Met-enkephalin or restraint stress on corticosterone release (fmol per mg protein in explants) from adrenal tissue *in vitro* in the presence or absence of naltrexone, adrenal corticosterone concentrations (fmol per mg protein in explants), and adrenal expression of PENK (expressed as a percentage of the mean vehicle treated pullets) in 14-week-old (sexually immature) female chickens (X ± SEM).

*In vivo* treatment	Corticosterone release from adrenal tissue *in vitro*		Adrenal corticosterone concentration	Adrenal PENK expression
	Basal	Naltrexone		
Vehicle	2.94 ± 0.31^ax^	8.0 ± 0.14^cy^	17.1 ± 0.19^c^	100 ± 1.86^b^
Met-enkephalin	4.78 ± 0.31^cx^	5.5 ± 0.32^by^	19.1 ± 0.13^d^	57.0 ± 1.48^a^
Stress	3.16 ± 0.16^abx^	4.4 ± 0.16^ay^	12.0 ± 0.32^b^	151 ± 1.83^d^
Met-enkephalin + stress	3.70 ± 0.24^bx^	5.0 ± 0.28^aby^	10.1 ± 0.22^a^	131 ± 6.52^c^
Two-way ANOVA F = (*p* =)				
Effect of met-enkephalin	**30.0 (5.04E** ^**-5**^ **)**	**13.3 (0.001)**	0.10 (0.758)	**77.1 (1.63E** ^**-7**^ **)**
Effect of stress	3.92 (0.065)	**76.0 (1.79E** ^**-7**^ **)**	**1,001 (7.44E** ^**-16**^ **)**	**299 (8.82E** ^**-12**^ **)**
Interaction	**8.96 (0.009)**	**41.5 (8.13** ^**E-6**^ **)**	**74.6 (2.03E** ^**-7**^ **)**	**10.3 (0.005)**

a, b, c, and d: Different superscript letters indicate difference (*p* < 0.05) with *in vivo* treatment (columns).

x and y: Different superscript letters indicate difference (*p* < 0.05) between basal *in vitro* release and that in the presence of naltrexone.

Bold indicates *p* < 0.05.

The effects of *in vivo* administration of Met-enkephalin or restraint stress on adrenal concentrations of corticosterone were less straightforward, with increases (*p* < 0.05) observed in pullets treated with Met-enkephalin, decreases (*p* < 0.001) in adrenal concentrations of corticosterone in stressed birds, and further decreases (*p* < 0.05) in stressed chickens receiving Met-enkephalin (Table 3).

**FIGURE 4:**
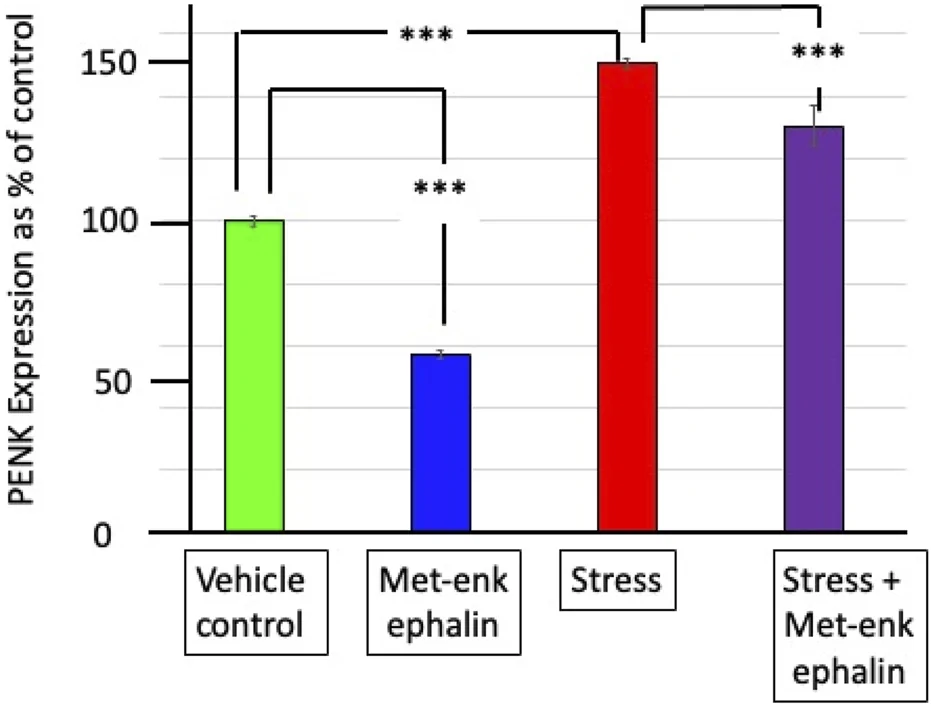
Effect of administration of Met-enkephalin or stress on PENK expression in the chicken adrenal glands (vertical lines indicate SEM). (*** indicates difference *p* < 0.001).

Administration of Met-enkephalin was followed by decreased (*p* < 0.001) expression of PENK in pullet adrenal tissue (Table 3; Figure 4). In contrast, restraint stress was followed by increased (*p* < 0.001) expression of PENK in chicken adrenal tissue (Table 3; Figure 4).

### 3.3 Relationships between circulating concentrations of corticosterone, Met-enkephalin, and total met-enkephalin together with adrenal corticosterone, PENK expression, and DOR binding

Putative relationships between circulating concentrations of corticosterone Met-enkephalin and peptides with Met-enkephalin motifs (excluding Met-enkephalin) were examined using the delta increase in concentrations as area under the curve minus initial, together with adrenal corticosterone, PENK expression, and DOR binding (Tables 4, 5). There was a very strong relationship (adjusted R^2^ > 0.9) between adrenal PENK expression and adrenal DOR binding (Table 4). There were strong relationships (adjusted R^2^ > 0.65) between plasma concentrations of corticosterone, as area under the curve minus initial, and both adrenal PENK expression and adrenal DOR binding (Table 4). There were also some relationships (*p* < 0.05) between and among plasma concentrations of corticosterone, native Met-enkephalin, total Met-enkephalin (Table 4), adrenal corticosterone concentrations, and release of corticosterone from adrenal explants in the presence or absence of naltrexone.

**TABLE 4:** Relationships between the increases/decreases in plasma concentration change as area under the curve and adrenal delta opioid binding and adrenal PENK expression.

Parameter	Adrenal delta opioid binding	PENK expression	Δ Plasma concentration change as area under the curve			
			Corticosterone	Native met-enkephalin	Total Met-enkephalin including native	Total met-enkephalin excluding native
Adrenal						
Delta opioid binding	1.00	**0.924 (*p* = 9.74E** ^**-12**^ **)** +3.50 ± 0.23	**0.670** (***p* = 6.33E** ^**-6**^) [+54.0 ± 8.7]	**0.365 (*p* = 0.003) [+35.7** ± **10.3]**	**0.398 (*p* = 0.002)** [-407 ± 111]	**0.400** (***p* = 0.002**) [−443 ± 120]
PENK expression		1.00	**0.736 (*p* = 8.00E** ^**-7**^ **) [+15.8** ± **2.15]**	**0.266 (*p* = 0.011) [+8.60** ± **3.06]**	**0.347 (*p* = 0.004) [-106** ± **31.7]**	**0.345 (*p* = 0.004) [**[−**111** ± **34.5]**
Δ Plasma concentration change as area under the curve						
Corticosterone			1.00	**0.409 (*p* = 0.001) [**−**6.22** ± **1.65]**	**0.241 (*p* = 0.016) [+0.45** ± **0.17]**	**0.399 (*p* = 0.002) [**−**6.67** ± **1.81]**
Native met-enkephalin				1.00	**0.852 (*p* = 4.26E** ^**-9**^ **) [10.2** ± **0.97]**	**0.874 (*p* = 9.73E** ^**-10**^ **) [11.2** ± **0.97]**
Total met-enkephalin including native					1.00	**0.999 (*p* = 3.54** ^**–** **27**^ **) [1.08** ± **0.008]**
Total met-enkephalin excluding native						1.00

Bold indicates statistically significant (*P* < 0.05).

**TABLE 5:** Relationships as adjusted R^2^ (*P* =) [slope ±SEM] between the adrenal parameters: delta opioid binding, PENK expression, *in vitro* corticosterone release in the presence or absence of naltrexone, corticosterone concentration in adrenal tissue, and the Δ plasma concentration change as area under the curve.

Parameters	Adrenal delta opioid binding	PENK expression	Corticosterone release *in vitro*—basal	Corticosterone release *in vitro*—naltrexone	Corticosterone in adrenal tissue	Δ plasma concentrations of corticosterone as area under the curve minus initial
Adrenal delta opioid binding	**1.0**	**0.924 (*P* = 9.74E** ^**-12**^ **) [+3.50** ± **0.23]**	**0.244 (0.016) [**−**0.045** ± **0.017]**	**0.177 (0.037) [**−**0.068** ± **0.030]**	**0.872 (1.00E** ^**-9**^ **) [**−**0.35** ± **0.03]**	**0.670** (***p* = 6.33E** ^**-6**^) **[+54.9** ± **8.7]**
PENK expression		1.0	**0.348 (0.004) [**−**0.014** ± **0.004]**	0115 (0.079)	**0.744 (6.07E** ^**-7**^ **) [**−**0.089** ± **0.012]**	**0.736 (*p* = 8.09E** ^**-7**^ **) [15.8** ± **2.15]**
Corticosterone release—basal			1.0	−0.005 (0.356)	0.075 (0.128)	**0.213 (0.023) [**−**398** ± **161]**
Corticosterone release—naltrexone				1.0	**0.269 (0.011) [+1.41** ± **0.50]**	**0.242 (0.016) [**−**241** ± **90.6]**
Corticosterone in adrenal tissue					1.0	**0.415 (0.0013) [**−**119** ± **31.3]**
						1.0

Bold indicates statistically significant (*P* < 0.05).

## 4 Discussion

In this study, plasma concentrations of corticosterone in chickens subjected to restraint were elevated (Table 1). This finding is consistent with other reports showing that immobilization stress rapidly increases plasma concentrations of corticosterone in chickens (Kadhim and Kuenzel, 2022; Scanes et al., 2024).

The distribution of DOR in chicken brains has been reported with expression in the hypothalamus, telencephalon, and midbrain (Bu et al., 2020). Intracerebroventricular administration of a DOR agonist stimulates feed intake in meat-type chickens (Bungo et al., 2004; 2005) but does not affect ghrelin depression of feed intake (Baghaeikia et al., 2022). In mammals, administration of dexamethasone *in vivo* is followed by decreases in the mu, delta, and kappa opioid receptor binding in the sheep adrenal cortex (Pierzchała-Koziec et al., 2015). Effects of delta, kappa, and mu opioid agonists on cortisol production from porcine adrenal cortical cells have been reported (Krazinski et al., 2011). Iyengar and colleagues (1987) concluded that delta opioid receptors play a role in the modulation of the HPA axis.

Synthetic opioids influence the release of glucocorticoids (cortisol in livestock and humans and corticosterone in rodents and poultry) and the HPA axis, with both stimulatory and inhibitory effects. Stimulatory effects include the following: administration of the Met-enkephalin analog, D-Ala^2^-Met-enkephalin amide, initially elevated but then depressed plasma concentrations of ACTH and corticosterone in rats (De Souza and Van Loon, 1982). Morphine induces increased plasma concentrations of corticosterone in rats (De Souza and Van Loon, 1982) and chickens (Scanes and Pierzchała-Koziec, 2024b). Moreover, peripheral, but not intracerebroventricular, administration of a delta opioid agonist was followed by increases in the plasma concentration of ACTH in fetal sheep (Taylor et al., 1997).

There are inhibitory effects of synthetic opioids. For instance, in humans, chronic opioid intake is associated with adrenal insufficiency (Donegan and Bancos, 2018; Li et al., 2020; reviewed by Coluzzi et al., 2023). Moreover, morphine depresses plasma concentrations of cortisol in humans (Zis et al., 1984; Zis et al., 1985). Furthermore, pre-treatment of sheep with morphine peripherally decreased the response to intracerebroventricular administration of corticotropin-releasing hormone (Parrott and Goode, 1992). Similarly, peripheral administration of the Met-enkephalin analog, [D-Ala^2^, N-Me-Phe^4,^ Met-(O)^5^-ol]-enkephalin (DAMME), decreased plasma concentrations of both cortisol and Met-enkephalin in sheep challenged with corticotropin-releasing hormone (CRH) (Redekopp et al., 1985). In this study, peripheral administration of the endogenous opioid, Met-enkephalin, to stressed or non-stressed birds was accompanied by decreased plasma concentrations of corticosterone in chickens (Figure 2; Table 1). Moreover, the increased *in vitro* release of corticosterone in the presence of the opioid receptor antagonist, naltrexone (Table 3), supports opioid inhibition of corticosterone production and release. In other studies, plasma concentrations of corticosterone were elevated in non-stressed chickens receiving the opioid antagonist naltrexone (Scanes and Pierzchala-Koziec, 2024a). Interestingly, in a similar manner, intracerebroventricular administration of Met-enkephalin to chicks was followed by reduced distress vocalizations, presumably reflecting lower levels of anxiety (Panksepp et al., 1978).

The differences between the effects of the endogenous opioid, Met-enkephalin (Table 1), and those of morphine (Scanes and Pierzchała-Koziec, 2024b) may reflect their relative ability to cross the blood–brain barrier, with morphine being able to stimulate the release of CRH and, consequently, ACTH and then corticosterone production and release. Although studies in rhesus monkeys indicate that Met-enkephalin can cross into the brain (Hartvig et al., 1986), the permeability of the blood–brain barrier to Met-enkephalin relative to morphine remains unclear. It is argued that peripheral Met-enkephalin predominantly, but not exclusively, affects the HPA downstream of the hypothalamus. In rats, a Met-enkephalin analog depresses *in vitro* production of corticosterone in response to ACTH (Guaza and Borrell, 1984). Moreover, *in vitro* release of cortisol and corticosterone from amphibian adrenal cortical cells was depressed by another endogenous opioid peptide, beta-endorphin (Zerani and Gobbetti, 1991; Zerani and Gobbetti, 1992). In addition, corticosterone release was increased in the presence of naloxone, presumably by mitigating the inhibitory effects of endogenous opioid peptides (Zerani and Gobbetti, 1991; Zerani and Gobbetti, 1992). There were increases in delta opioid agonist binding to cell membranes in pullets subjected to restraint stress and, consequently, plasma concentrations of corticosterone (Table 5; Figure 4). To the best of our knowledge, this is the first report of stress augmenting delta opioid receptors. The increases in delta opioid agonist binding to cell membranes are consistent with the hypothesized cross-talk between the HPA axis and opioid peptides (Table 5; Figure 4). Furthermore, it is argued that with increased delta opioid agonist binding, there would be increased efficacy of Met-enkephalin and other opioid peptides.

The decreases in delta opioid agonist binding (Figure 4; Table 4) to membranes in pullets injected with Met-enkephalin are readily explicable by downregulation/internalization *in vivo*. There is substantial evidence that opioids induce downregulation of opioid receptors, particularly delta opioid receptors, in neural tissue. For instance, prolonged morphine treatment also induces augmentation of post-internalization trafficking of delta opioid receptors (Ong et al., 2015). Furthermore, the pentapeptide Met-enkephalin induced downregulation of delta opioid receptors in neuroblastoma–glioma NG108-15 hybrid cells (Simantov et al., 1982). Moreover, in a similar manner, mu-opioids induced internalization of both mu- and delta-opioid receptors (Bao et al., 2018).

To the best of our knowledge, this is the first report of Met-enkephalin decreasing its own synthesis and, perhaps, also its release in any species. It remains unclear why total Met-enkephalin levels in the circulation (Table 3), together with adrenal expression of PENK (Figure 3; Table 3), were decreased in pullets receiving Met-enkephalin i.v. These are consistent with Met-enkephalin exerting a “negative-feedback”-like effect. In a similar manner, the opioid agonist, morphine, reduced both plasma concentrations of Met-enkephalin and adrenal PENK expression (Scanes and Pierzchała-Koziec, 2024b).

It remains unclear what total Met-enkephalin reflects. Possible contributors include partially processed pro-enkephalin or extended biologically active peptides containing Met-enkephalin motifs or carrier proteins that chaperone Met-enkephalin. It is recognized that a second biologically active neuropeptide is present in pro-enkephalin, namely, Leu-enkephalin.

## 5 Conclusion

Peripheral injection of exogenous Met-enkephalin depressed the HPA axis activity during stress by decreasing the adrenal corticosterone release and adrenal PENK expression while unexpectedly attenuating the stress-induced increase in delta receptor binding in the pituitary and adrenal glands. It is suggested that endogenous Met-enkephalin modulates the activity of the HPA axis during the stress response by acting at central and peripheral levels.

## Data Availability

The raw data supporting the conclusions of this article will be made available by the authors, without undue reservation.
