# Correction: Met-enkephalin modulates the stress responses of plasma concentrations of corticosterone, delta opioid receptor binding, pro-enkephalin expression, and processing in chickens

**DOI:** 10.3389/fphys.2026.1936355

**Published:** 2026-08-07

**Authors:** Krystyna Pierzchała-Koziec, Colin G. Scanes, Klaudia Jaszcza

**Affiliations:** 1 Department of Animal Physiology and Endocrinology, University of Agriculture in Krakow, Krakow, Poland; 2 Department of Biological Science, University of Wisconsin Milwaukee, Milwaukee, WI, United States

**Keywords:** chicken, corticosterone, Met-enkephalin, pro-enkephalin, stress response

There was a mistake in **Figure 1** as published. Instead of **Figure 1** the **Figure 2** was published twice. The corrected **Figure 1** appears below.

**Figure 1:**
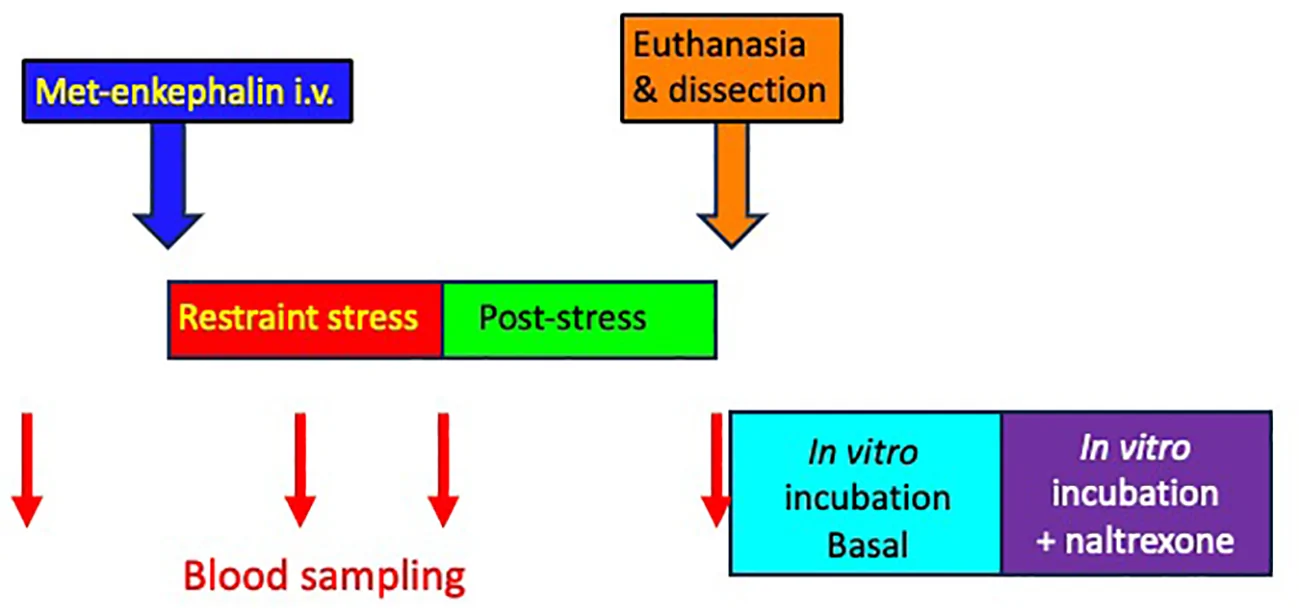
Schematic representation of the experimental design.

The original version of this article has been updated.

